# In Memoriam

**Published:** 1880-09

**Authors:** 


### IN MEMORIAM.

" Died at Salem, Mass., August 1st, 1880, of Chronic Bright's Disease, Porteus P. Bielby, M. D., Assistant-Surgeon U. S. Navy."

The above, clipped from the secular press, conveys the announcement of the early death of one of the most promising of the junior members of the profession of this city. The event, long anticipated, has cast a gloom over the social and professional circles here and elsewhere, in which his bright mind, and genial and manly nature have been the passport to strong personal friendships.

Dr. Bielby was born in Little Falls, N. Y., Feb. 24, 1846. Receiving his preparatory training in the academy of his native town, he entered the University of Toronto at the early age of fifteen years, where he remained for two years, giving up his college course to enter the army, in which he served as first lieutenant and afterwards as adjutant. At the close of the war he commenced the study of medicine, graduating at the Buffalo Medical College in the class of 1868. Soon after he appeared before the Naval Medical Board and passed second in a class of forty, and received his commission as assistant-surgeon. On his first voyage, from exposure in a severe storm on the South American coast, he contracted the disease which led to his death.

This brief outline of his life shows how rapid had been his preferment, reaching an honorable position in the government service at an unusually early age, and acquiring an enviable reputation for scientific and professional attainments in the public service as well as in private practice. He excelled also in the possession of social qualities, which attracted to him a large circle of warm admirers. He was always the bright and genial companion, the courteous gentleman, the honorable citizen.

We regard the death of Dr. Bielby as a great loss to the medical profession, in which his acknowledged ability would have found a broad field for exercise, gathering results by industry, which would have secured for him a yet higher position and wider reputation. He fought bravely against the almost inevitable fate, which attends this disease, traveling extensively to alleviate his sufferings or to ameliorate the physical infirmity which had befallen him. His efforts proving futile, he awaited with all the true manliness of his noble character the approach of death, strong in his religious convictions, and conscious that in his life he had utilized the rare gifts of mind and heart with which he was endowed, in the service of the most humane of the learned professions.

